# Supplementary figures and images for: Mapping and Screening of Candidate Gene Regulating the Biomass Yield of Sorghum (Sorghum bicolor L.)
Source: Int J Mol Sci. 2024 Jan 8;25(2):796. doi: 10.3390/ijms25020796 (PMC10815252; doi:10.3390/ijms25020796)

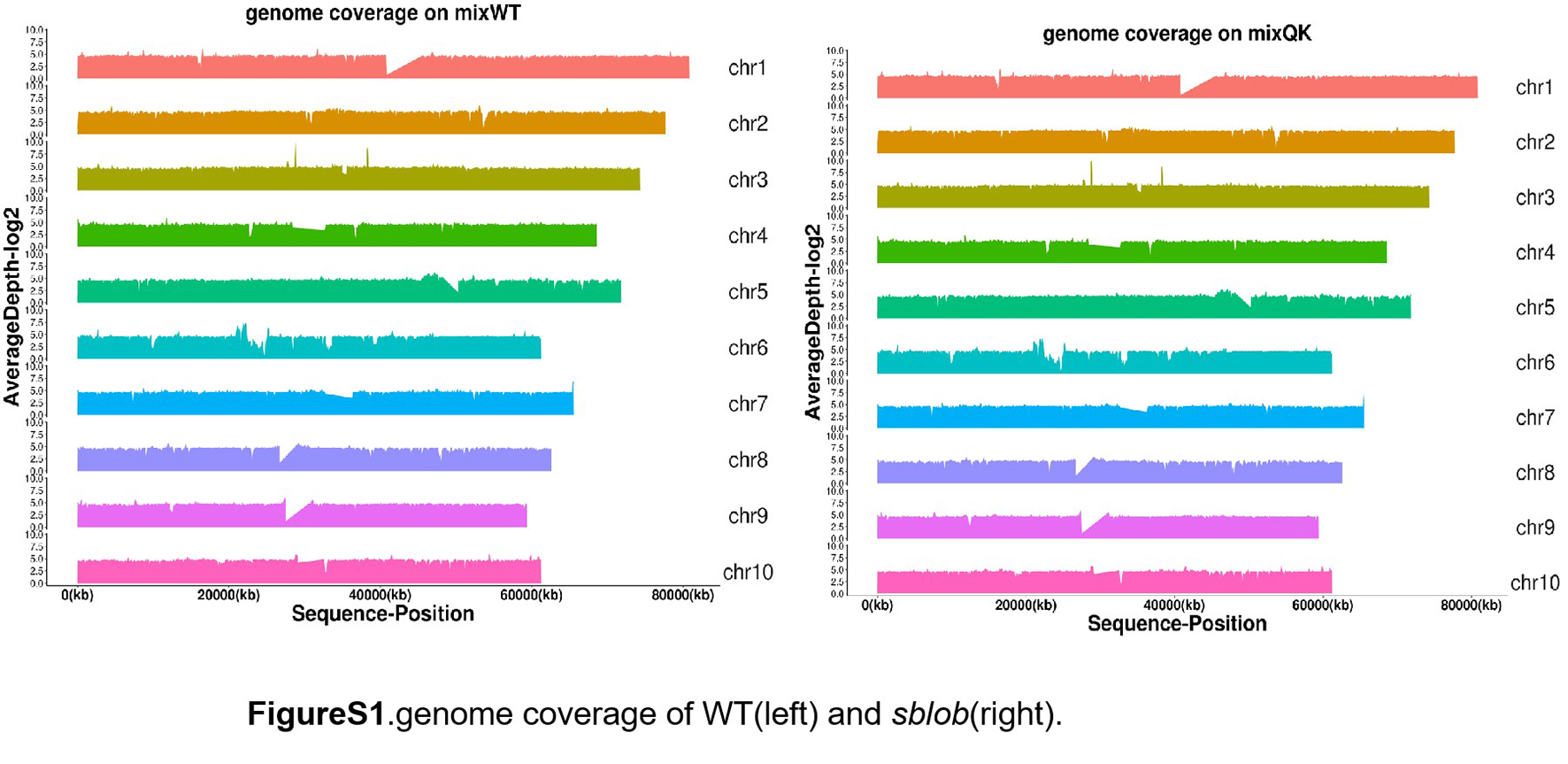

Supplement: Supplementary file 1 [file ijms-25-00796-s001.zip › figureS1.jpg]

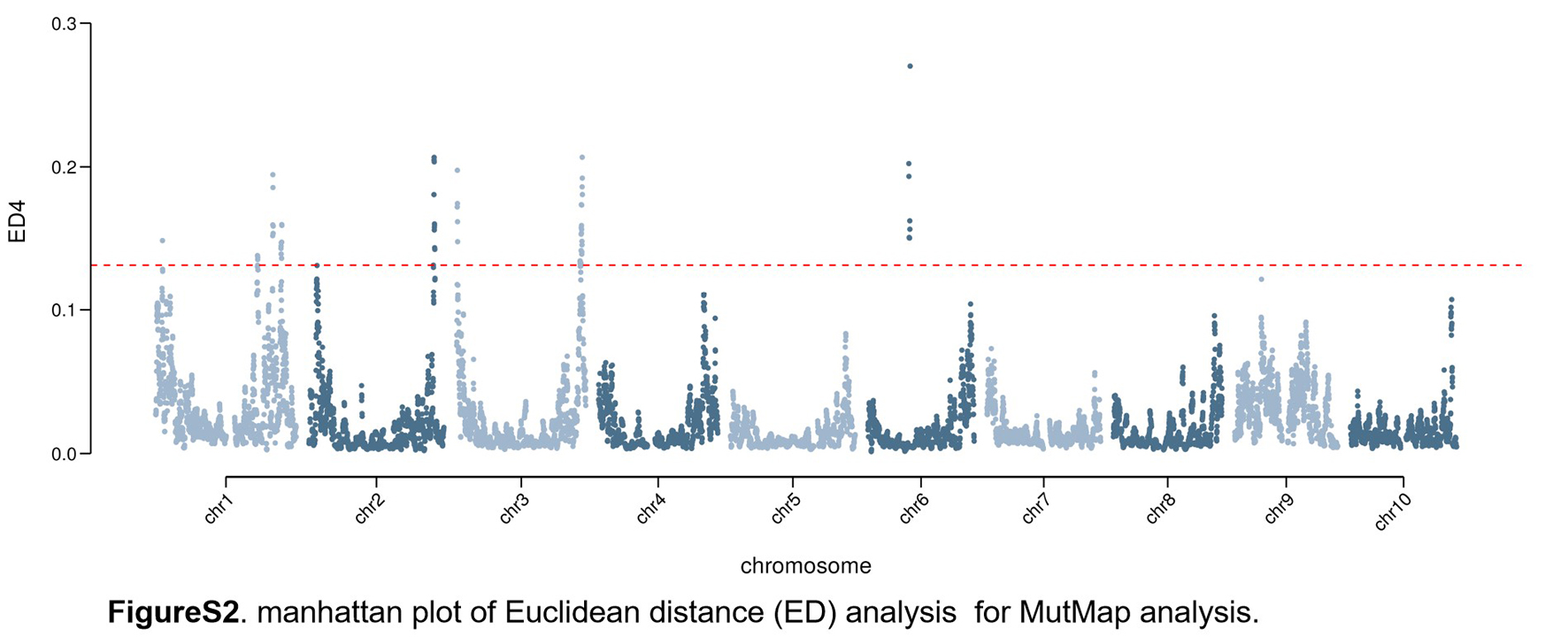

Supplement: Supplementary file 1 [file ijms-25-00796-s001.zip › figureS2.jpg]

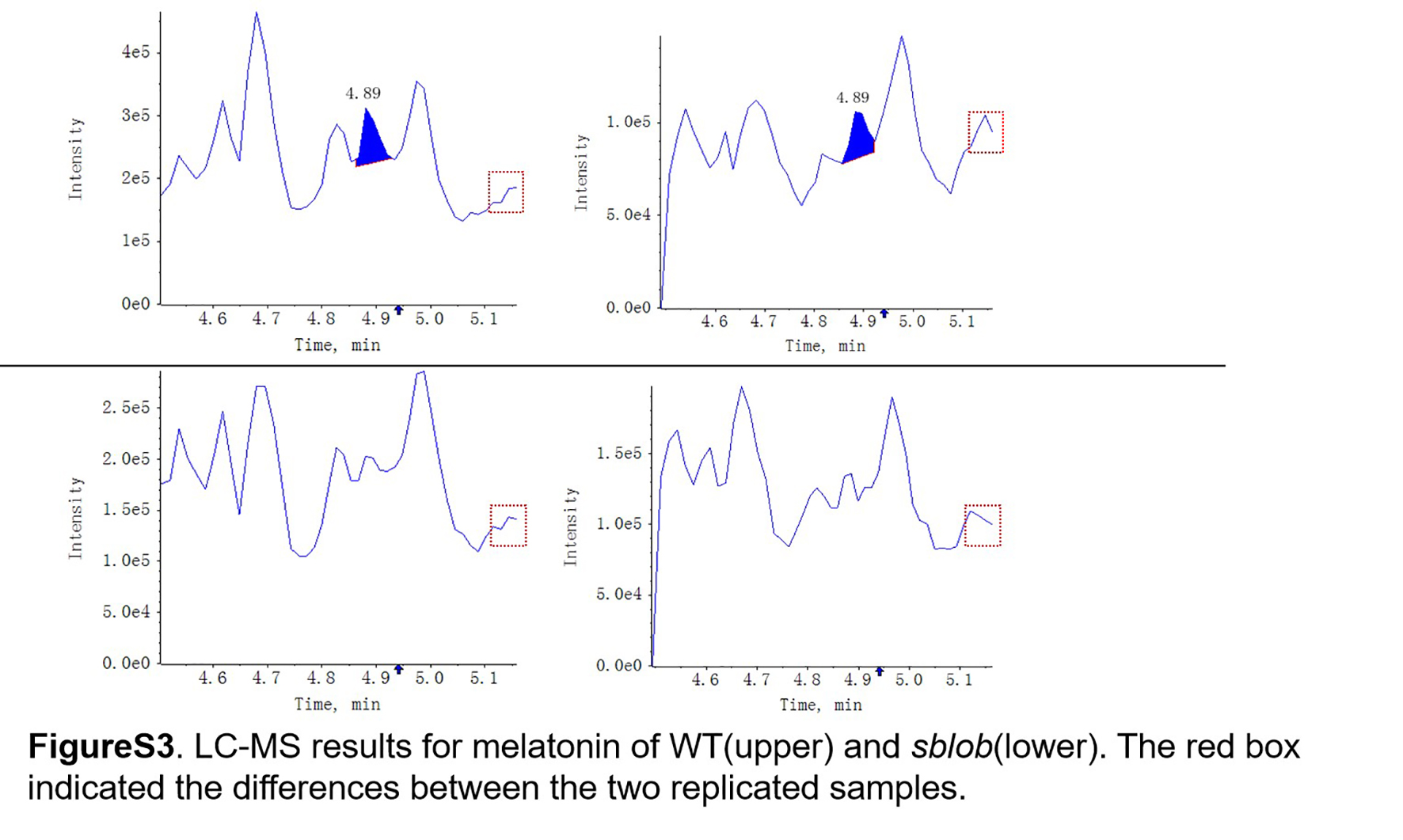

Supplement: Supplementary file 1 [file ijms-25-00796-s001.zip › figureS3.jpg]

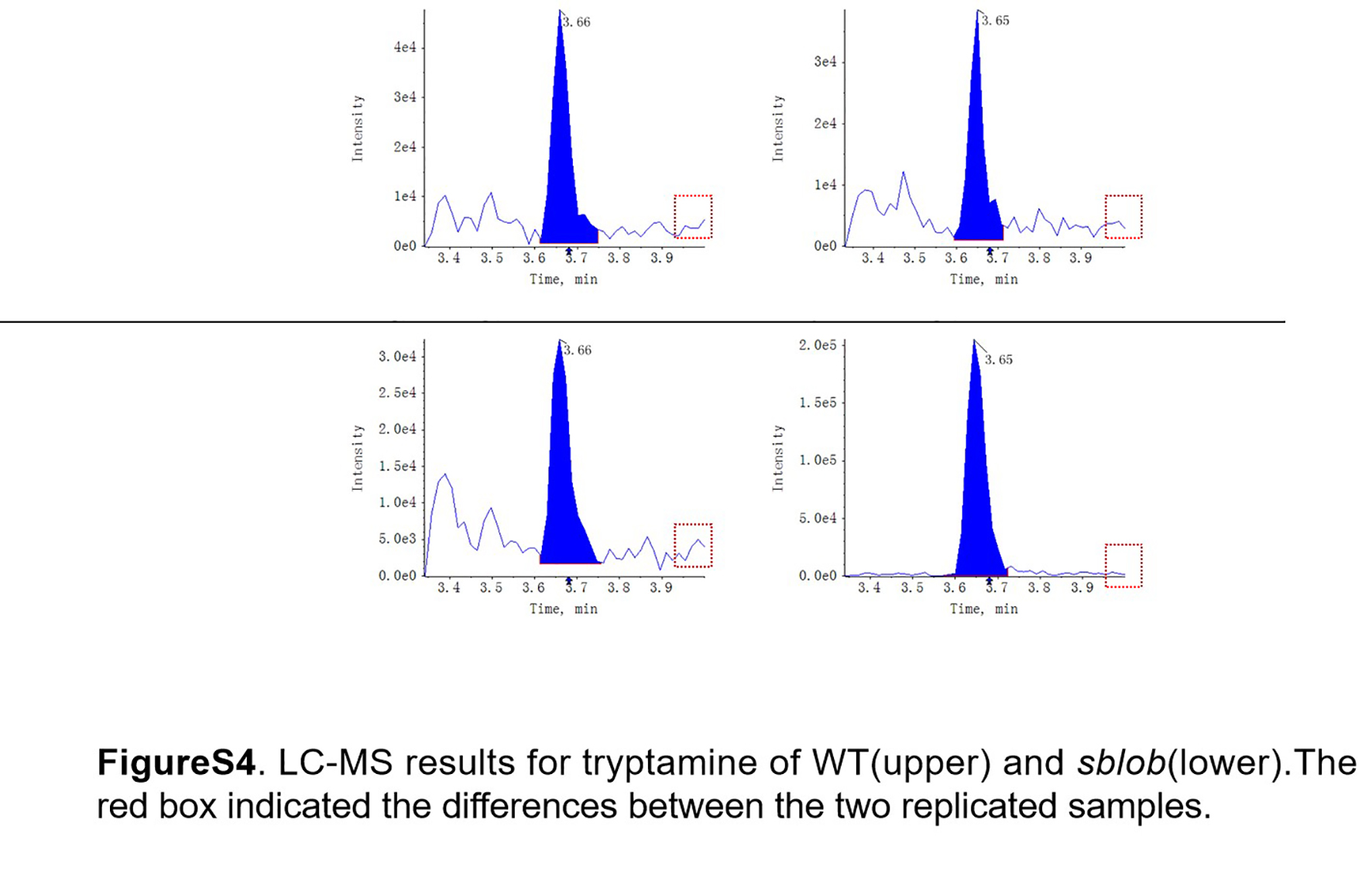

Supplement: Supplementary file 1 [file ijms-25-00796-s001.zip › figureS4.jpg]

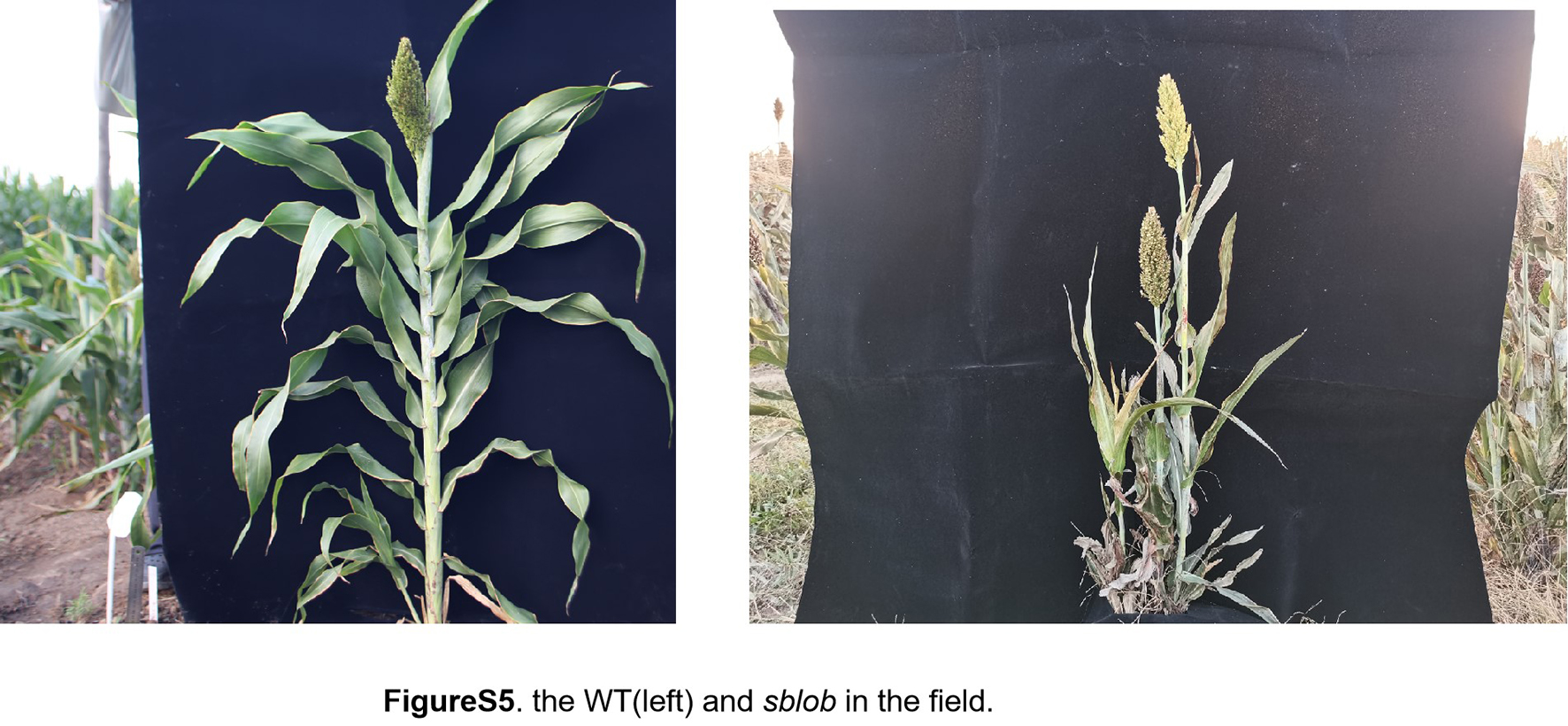

Supplement: Supplementary file 1 [file ijms-25-00796-s001.zip › figureS5.jpg]
